# KOH-activated carbon from pomelo peels for CO_2_ adsorption: influence of activation and adsorption process parameters

**DOI:** 10.1007/s11356-026-37642-x

**Published:** 2026-03-25

**Authors:** Nawal Abd Ghafar, Nor Ruwaida Jamian, Yuki Nagao, Kentaro Aoki, Eleen Dayana Mohamed Isa, Grace Erlinda Harimisa, Nurfatehah Wahyuny Che Jusoh

**Affiliations:** 1 Department of Chemical and Environmental Engineering (ChEE), Malaysia-Japan International Institute of Technology (MJIIT), Universiti Teknologi Malaysia, Kuala Lumpur, Malaysia; 2 School of Materials Science, Japan Advanced Institute of Science and Technology, 1–1 Asahidai, Nomi, Ishikawa 923-1292 Japan; 3 Center of Hydrogen Energy, Institute of Future Energy, Universiti Teknologi Malaysia, Kuala Lumpur, Malaysia

**Keywords:** CO_2_ capture, Pomelo peel, KOH activation, Activation parameters, Adsorption parameters, Response surface methodology

## Abstract

This study examines pomelo peel-based activated carbon (PMAC) as a promising and sustainable carbon dioxide (CO_2_) adsorbent through a systematic exploration of the influence of activation and adsorption process parameters. Investigations into the effects of the activation parameters revealed that the best potassium hydroxide (KOH) activation conditions were achieved with a KOH to pomelo peel-based char mass ratio of 3:1, an activation time of 1.5 h, and an activation temperature of 700 °C. The resulting activated carbon, designated as PMAC-3-1.5-7 (where 3, 1.5, and 7 represent the KOH ratio, activation time in hours, and temperature in hundreds Celsius, respectively), exhibited the highest CO_2_ uptake of 254.3 mg/g at 25 °C and 30 bar. Additionally, the response surface methodology (RSM) optimisation method applied to the adsorption process parameters indicated that the maximum adsorption capacity (203 mg/g) occurred at an adsorption temperature of 25 °C, a pressure of 30 bar, and a 50% CO_2_ composition in the CO_2_-N_2_ gas mixture within the parametric study. These findings demonstrate the potential of pomelo peel-based activated carbon as a sustainable and effective adsorbent for CO_2_ capture, offering valuable insights into the effects of activation and adsorption process parameters on its CO_2_ adsorption performance.

## Introduction

Continuous carbon dioxide (CO_2_) emission to the atmosphere is immensely concerning due to the critical environmental issues it poses, specifically global warming and climate change. According to the National Oceanic and Atmospheric Administration (NOAA)’s Global Monitoring Lab, the global average atmospheric CO_2_ concentration in 2024 is measured at 422.9 ppm, which is significantly higher than 390.5 ppm in 2011 (Lan et al. 2024). To reduce CO_2_ emissions to the atmosphere, carbon capture and storage (CCS) technology has been explored and implemented in carbon-intensive industries such as fossil-fuelled power plants. Amine-based scrubbing, which utilises amine-based solvents, such as monoethanolamine (MEA) and diethanolamine (DEA) to absorb CO_2_ from the gas stream, is widely regarded as the most mature method for CO_2_ capture in the industry (Peyravi 2018). This method, however, has its own shortcomings, which include solvent degradation, equipment corrosion, and high solvent regeneration energy (Chen et al. 2016).

Adsorption is deemed the most promising method to capture CO_2_ due to its simplicity, energy efficiency in terms of adsorbent regeneration, and lower corrosion potential to the equipment. The effectiveness of the adsorption process depends on the adsorbent properties. Various adsorbents have been researched for CO_2_ adsorption, such as activated carbon, zeolites, metal-organic frameworks, silica, and metal oxides (Kenarsari et al. 2013; Sun et al. 2018; Zhao et al. 2018a; Lian et al. 2019). Amongst these adsorbents, activated carbon is highly favourable because it is inexpensive, relatively hydrophobic, non-corrosive, generally requires lower regeneration energy, and possesses high porosity (Plaza et al. 2014; Zhang et al. 2019). The investigation of biowaste as a precursor for activated carbon has been receiving considerable attention in recent years due to its cost-effective and sustainable approach. This strategy can also positively contribute to the waste management issue by reducing landfill disposal and converting waste into a valuable product. Recently, several studies conducted on biowastes as an activated carbon precursor for CO_2_ adsorption are tea seed shell (Quan et al. 2020a), rice husk (He et al. 2021), walnut shell (Guo et al. 2022), water chestnut shell (Ma et al. 2022), and macadamia nut shell (Bai et al. 2023). Pomelo peels are ideal biowaste precursors for activated carbon due to their high carbon and volatile matter content, which contribute to pore formation within the material (Chen et al. 2020; Zhao and Chen 2020). Pomelo is amongst the most abundant citrus fruits, with over 9 million tonnes produced worldwide, and the peels constitute around 30% to 50% of the total fruit weight (Looyrach et al. 2015; Xiang et al. 2024). The high porosity of activated carbon derived from pomelo peel has led to its use for several research purposes, including supercapacitors (Yang et al. 2024) and adsorbents for heavy metals and dyes (Zhao et al. 2018b; Zhang et al. 2022).

Previous literature has demonstrated that activation process parameters play a crucial role in tailoring activated carbon to achieve desirable properties for high CO_2_ adsorption performance. For instance, Serafin et al. (2022) studied the effect of activation temperature on surgical mask-derived activated carbon and discovered that 800 °C is the optimal activation temperature for CO_2_ adsorption capacity at ambient conditions due to its narrow pore size distribution. Additionally, Ko et al. (2017) synthesised a series of mesoporous carbons at various activation temperatures and activation agent mass ratios, showing that the sample with the highest surface area and mesopore volume exhibited the best CO_2_ adsorption performance. Furthermore, Kaur et al. (2019) found that varying the activation time, activation temperature, and activation agent ratio produced PET-based activated carbon with the highest microporosity, resulting in excellent CO_2_ adsorption performance at 25 °C and 1 bar. Given the impact of activation process parameters, especially on the textural properties of activated carbon, it is therefore necessary to investigate the impacts of these parameters on the physicochemical properties of activated carbon and its CO_2_ adsorption performance. Past studies have primarily conducted CO_2_ adsorption performance analysis of activated carbons at atmospheric pressure, with insufficient studies across a range of both low and high pressures. This aspect is important as it explores the applicability for both post-combustion and pre-combustion applications, which typically operate at atmospheric and high pressures, respectively.

Besides the activation parameters, the influence of adsorption process parameters is also considered paramount to the CO_2_ adsorption performance of the adsorbent. A study by Azharul Islam et al. (2018) employed response surface methodology (RSM) to optimise adsorption temperature, adsorbent loading, and CO_2_ composition to produce a chitosan-bleaching earth clay composite adsorbent with maximum CO_2_ adsorption capacity. Similarly, adsorption process parameters, such as CO_2_ composition and temperature, were also investigated and optimised by Thouchprasitchai et al. (2018) to attain maximum CO_2_ adsorption capacity of the tetraethylenepentamine-modified hydrotalcite. Furthermore, Khajeh and Ghaemi (2021) included both adsorption temperature and pressure amongst the parameters that were investigated and optimised for the CO_2_ adsorption process of strontium hydroxide-modified nanoclay montmorillonite. According to available literature, specifically on CO_2_ adsorption of activated carbon, there are apparently limited studies that investigate and optimise CO_2_ adsorption process parameters using statistical tools like RSM.

In this study, a series of pomelo peel-based activated carbon was synthesised through chemical activation using potassium hydroxide (KOH) as an adsorbent for CO_2_ capture. The effects of potassium hydroxide (KOH) activation parameters such as activation time, activation agent ratio, and activation temperature were investigated on the physicochemical properties of the pomelo peel-based activated carbon and its CO_2_ adsorption performance across a wide pressure range (0 to 30 bar). Additionally, the adsorption process parameters such as adsorption temperature, pressure, and CO_2_ composition were investigated and optimised for the pomelo peel-based activated carbon by using RSM modelling.

## Materials and methods

### Materials

The peels of the pomelo fruits were obtained from a local fruit store in Shah Alam, Selangor, Malaysia. The pellets of potassium hydroxide (KOH) and hydrochloric acid (HCl) of analytical reagent grade were bought from R&M Chemical Sdn. Bhd. Additionally, 99.999% of purified gases, including argon, carbon dioxide, nitrogen, and helium, were supplied from Alpha Gas Solutions Malaysia.

### Synthesis of adsorbent

#### Carbonisation of pomelo peel

The entire pomelo peels (PM), encompassing both white mesocarp and green exocarp, were washed repeatedly with distilled water to remove dirt and impurities from the surface, followed by 24 h oven dried at 110 °C. Subsequently, the dried pomelo peels were carbonised inside a tubular furnace (Keijia), with argon gas continuously flowing through the quartz tube at 600 °C for 2 h to produce the pomelo peel-based char (PMC).

#### Chemical activation of pomelo peel-based biochar

The PMC underwent a chemical activation process by using KOH as the activation agent. Firstly, the PMC was impregnated with KOH solution at 80 °C for 6 h at various KOH to PMC mass ratios (2:1, 3:1, and 4:1). The samples were then oven dried at 110 °C for 24 h. Following that, the impregnated material was activated inside the tubular furnace under continuous argon gas flow at different activation times (1 h, 1.5 h, and 2 h) and temperatures (500 °C, 600 °C, and 700 °C). Subsequently, the samples were washed with 0.1 M HCl and distilled water to achieve a pH of 7 in the filtrate. To minimize the presence of residual salts that may interfere with pore accessibility and adsorption performance, multiple sequential washing cycles procedures can be applied to ensure more complete removal of salt residues.

**Fig. 1:**
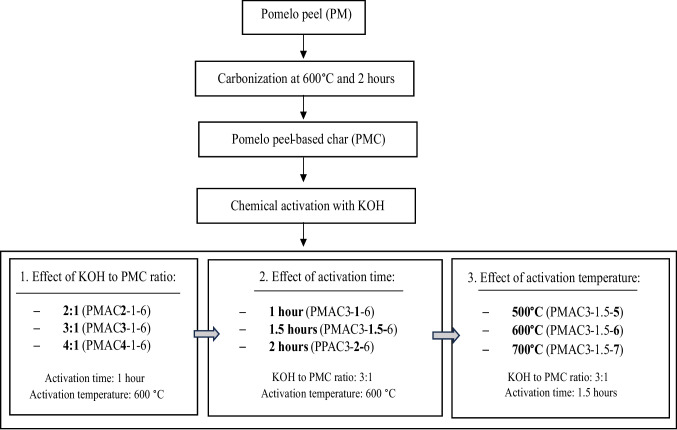
The process flowchart for the synthesis of PMAC

The pomelo peel-based activated carbon (PMAC) was finally produced after oven dried at 110 °C for 24 h. The one-factor-at-a-time (OFAT) approach was implemented in this study to determine the best activation parameters for producing PMAC, with porosity (BET surface area and total pore volume) as the response variable. The designated name of the samples is PMACx-y-z, where *x* represents the KOH to PMC ratio, *y* represents activation time, and *z* represents the first digit of the activation temperature. Figure 1 depicts the overall flowchart for the synthesis of PMAC, and Table 1 presents the sample names of PMAC based on the effects of the activation parameters.

**Table 1:** Sample names of PMAC based on the effects of activation parameters

Sample name	Activation agent ratio (KOH:PMC)	Activation time (h)	Activation temperature (°C)
1. **Effect of activation agent ratio**			
PMAC2-1–6	2:1	1	600
PMAC3-1–6	3:1	1	600
PMAC4-1–6	4:1	1	600
2. **Effect of activation time**			
PMAC3-1–6	3:1	1	600
PMAC3-1.5–6	3:1	1.5	600
PMAC3-2–6	3:1	2	600
3. **Effect of activation temperature**			
PMAC3-1.5–5	3:1	1.5	500
PMAC3-1.5–6	3:1	1.5	600
PMAC3-1.5–7	3:1	1.5	700

### Characterisation

The pore attributes of the samples were analysed through nitrogen (N_2_) adsorption-desorption at −196 °C by using Micromeritics ASAP 2020. The specific surface area and average pore diameter were calculated by using the Brunauer-Emmett-Teller (BET) method. The total pore volume was obtained from the N_2_ adsorption-desorption at relative pressure of 0.99. The micropore volume and pore size distribution were determined using the t-plot and density functional theory (DFT) methods, respectively. Fourier transform infrared spectroscopy (FTIR) analysis was conducted on the samples by using the Shimadzu IR-Tracer100 with the potassium bromide (KBr) pellet method. Surface functional groups were identified from the recorded FTIR spectra in the 500 to 4,000 cm^−1^ wavenumber region. The crystallinity of the samples was examined using the X-Ray Diffractometer, Empyrian Series 2 model, emitting 40 mA and 40 kV CuKα radiation to produce a diffraction pattern within the 5° < 2*θ* < 90° region. The thermal stability of the samples was evaluated using the Tru Mass™ balance (TGA55) through continuous heating from 30 to 1000 °C under N_2_ gas flow at 10 °C/min. The surface morphology of the samples was examined through scanning electron microscopy (SEM) analysis, using a JEOL JSM-5T300 at 10.0 kV and a Hitachi TM3030 Plus at 15.0 kV, to generate images of the sample surfaces.

### Gravimetric adsorption measurement

The gas adsorption measurement was conducted using a Rubotherm Isosorp Gravimetric Analyser, which integrates a magnetic suspension balance (MSB) to precisely measure the mass change of the adsorbent before and after gas adsorption; this method is referred to as the gravimetric adsorption method. To obtain the adsorption isotherm of the adsorbents, three steps were performed: pretreatment, buoyancy measurement, and adsorption. The pretreatment step involved degassing the adsorbents at 80 °C under vacuum conditions to ensure the complete removal of moisture and impurities from the surface. Following that, the buoyancy measurement step was conducted in helium gas to precisely measure the adsorbent’s mass and volume. The adsorption step was then carried out with the equilibrium adsorption capacity measured at each pressure interval. For the PMAC adsorbents synthesised at various activation parameters, the adsorption measurement was carried out at 25 °C, pressure from 0 to 30 bar, and pure CO_2_ gas feed. Equation 1 and Eq. 2 were utilised by the instrument to calculate the adsorption capacity of the adsorbent. It is noteworthy that the computation for the mass of the adsorbed gas is determined from the equilibrium of forces (gravitational force, buoyancy force, force from gas adsorption) within the MSB.

1 $$\text{Mass of the adsorbed gas}, {m}_{\text{AG }}(\mathrm{g})={m}_{\mathrm{MSB}}-{m}_{\mathrm{AC}}-{m}_{\mathrm{A}}+\left({V}_{\mathrm{AC}}+{V}_{\mathrm{A}}+{V}_{\mathrm{AG}}\right){\rho }_{\mathrm{G}}$$

2 $$\text{Adsorption capacity }\left(\mathrm{mg}/\mathrm{g}\right)=\frac{{m}_{\mathrm{AG}}}{{m}_{\mathrm{A}}} \times 1000$$

where *m*_MSB_ (g) is the mass reading from the MSB, *m*_AC_ (g) is the mass of the adsorbent container, *m*_A_ (g) is the mass of the adsorbent, *V*_AC_ (cm^3^) is the volume of the adsorbent container, *V*_A_ (cm^3^) is the volume of the adsorbent, *V*_AG_ (cm^3^) is the volume of the adsorbed gas, and *ρ*_G_ (g/cm^3^) is the gas density.

### RSM experimental design for adsorption process parameters

The investigation of the effects of CO_2_ adsorption process parameters on the adsorption capacity of the adsorbent, and the optimisation of these parameters, was carried out by using a response surface methodology (RSM) approach. The independent variables in the RSM experimental design are adsorption temperature, pressure, and CO_2_ composition (in CO_2_ and N_2_ gas mixture), with the response variable being the adsorption capacity measured by the Rubotherm Isosorp Gravimetric Analyser. The levels of these independent variables are detailed in Table 2. The selection of parameter values is based solely on previous studies that utilised RSM optimisation for CO_2_ adsorption process parameters (Saeidi et al. 2018, 2020; Thouchprasitchai et al. 2018; Khajeh and Ghaemi 2021).

**Table 2:** Parameters and level for the experimental design

Independent variable	Coded variable levels		
	−1	0	1
Temperature (°C)	25	45	65
Pressure (bar)	10	20	30
CO_2_ composition (%)	10	30	50

The Box-Behnken design (BBD) was selected for the experimental design over the frequently used central composite design (CCD) because BBD does not include points outside the defined ranges, which are present in CCD due to its inclusion of axial points (Szpisják-Gulyás et al. 2023). This prevents the design points from falling into non-operational or impractical settings. Moreover, for three factors, fewer experimental runs are required for BBD as compared to CCD, which can save time, resources, and improve efficiency. BBD is a 3-level design that requires 17 experiments, as opposed to 19 experimental runs for CCD with 5 centre points. The experimental runs were randomised to eliminate the effect of uncontrollable factors. Expert Design v13.0, a statistical software, was utilised to design the experiment, generate a predictive empirical model, statistically analyse the model and parameter effects, and optimise the parameters to maximise the adsorption capacity of the adsorbent.

## Results and discussion

### Characterisation

#### N_2_ adsorption-desorption analysis

The pore properties of raw pomelo peel (PM) and pomelo peel-based activated carbons (PMAC) were characterised through N_2_ adsorption-desorption at −196 °C. The resulting pore property values are given in Table 3. From Table 3, the significantly higher surface area value of all PMACs compared to PM (1.5 m^2^/g) demonstrates the presence of abundant pores in PMACs due to the carbonisation and KOH activation process. The carbonisation process contributed to the initial formation of pores through the thermal decomposition of volatile organic components in PM. Subsequently, the pores were further developed from the KOH activation process, whereby it potentially removed pore blockages in PMC caused by tar and other carbonaceous deposits, while generating new pores through gasification, reduction reactions of potassium oxide and carbonate (K_2_O and K_2_CO_3_), and subsequent potassium metal intercalation into the carbon matrix (Nowrouzi et al. 2017; Kaur et al. 2019). Equation 3 to Eq. 8 are the chemical reactions involved in the KOH activation process according to previous studies (Song et al. 2013; Kaur et al. 2019; Quan et al. 2020b).

**Table 3:** Pore properties of PM and PMACs and pure CO_2_ adsorption performance of PMACs

Sample	Surface area (m^2^/g)	Total pore volume (cm^3^/g)	Micropore volume (cm^3^/g)	Average pore diameter (nm)	CO_2_ adsorption at 25 °C (mg/g)		
					1 bar	10 bar	30 bar
PM	1.5	-	-	4.94	-	-	-
**i) Effect of KOH to PMC ratio (2:1, 3:1, 4;1)**							
PMAC**2**−1−6	170.5	0.09	0.07	2.16	103.8	154.8	175.7
PMAC**3**−1−6	386.4	0.20	0.13	2.04	118.7	189.6	218.7
PMAC**4**−1−6	383.7	0.19	0.13	2.00	112.1	176.9	186.8
**ii) Effect of activation time (1 h, 1.5 h, 2 h)**							
PMAC3-**1**−6	386.4	0.20	0.13	2.04	118.7	189.6	218.7
PMAC3-**1.5**−6	392.4	0.21	0.14	2.13	127.1	206.3	234.6
PMAC3-**2**−6	356.9	0.18	0.12	2.06	123.7	197.3	208.1
**iii) Effect of activation temperature (500 °C, 600 °C, 700 °C)**							
PMAC3-1.5-**5**	302.4	0.17	0.12	2.21	109.0	169.9	196.7
PMAC3-1.5-**6**	392.4	0.21	0.14	2.13	127.1	206.3	234.6
PMAC3-1.5**−7**	455.5	0.24	0.16	2.07	128.7	214.3	254.3

3 $$2\mathrm{KOH}\to {\mathrm{K}}_{2}\mathrm{O}+{\mathrm{H}}_{2}\mathrm{O}$$

4 $$\mathrm{C}+{\mathrm{H}}_{2}\mathrm{O}\to {\mathrm{H}}_{2}+\mathrm{CO}$$

5 $$4\mathrm{KOH}+\mathrm{C}\to {\mathrm{K}}_{2}\mathrm{O}+3{\mathrm{H}}_{2}+{\mathrm{K}}_{2}{\mathrm{CO}}_{3}$$

6 $${\mathrm{K}}_{2}{\mathrm{CO}}_{3}\to {\mathrm{K}}_{2}\mathrm{O}+{\mathrm{CO}}_{2}$$

7 $${\mathrm{K}}_{2}{\mathrm{CO}}_{3}+2\mathrm{C}\to 2\mathrm{K}+3\mathrm{CO}$$

8 $${\mathrm{K}}_{2}\mathrm{O}+\mathrm{C}\to 2\mathrm{K}+\mathrm{CO}$$

**Fig. 2:**
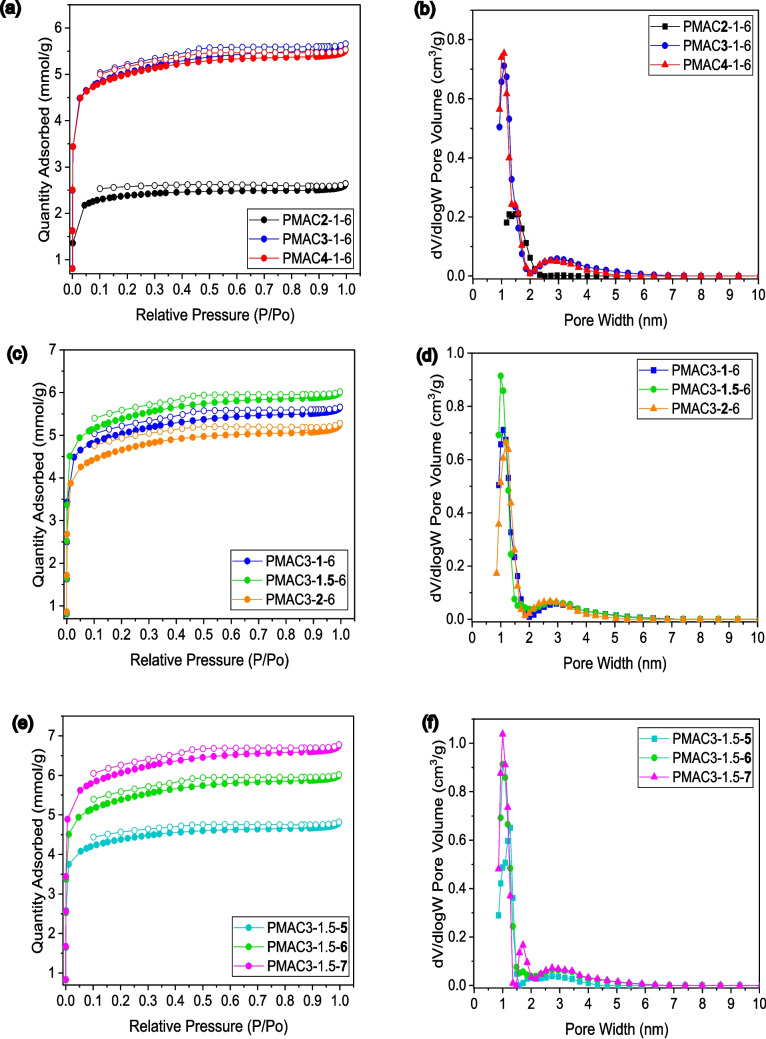
N_2_ adsorption–desorption isotherm and pore size distribution of PMACs synthesised under various (**a**-**b**) KOH to PMC ratio (**c**-**d**) activation time (**e**–**f**) activation temperature

Figure 2 shows the N_2_ adsorption/desorption isotherm and pore size distribution plots of the PMACs synthesised under different activation parameters (KOH to PMC mass ratio, activation time, and activation temperature). The N_2_ adsorption/desorption isotherm plots in Fig. 2a, c, and e exhibited similar isotherm shapes for all PMACs—type I(b), with minimal H4 loop hysteresis. The observed sharp increase in adsorption capacity at low relative pressure (*P*/Po < 1) indicates micropores filling, whereas a broader knee of the isotherm highlights the existence of larger-sized micropores and mesopores in the structure. The small hysteresis loop also validates the presence of mesopores (Liu and Huang 2018). The pore size distribution plots in Fig. 2b, d, and f displayed the pores of all PMACs mainly concentrated within a pore width region less than 2 nm (i.e. micropores), with a lesser volume of pores existing between 2 and 6 nm. All of these observations indicate that the internal structure of PMACs is microporous-mesoporous.

In order to investigate the effect of the activation agent ratio (KOH to PMC mass ratio) on the pore structure of PMACs, three separate tests were carried out at a constant activation time of 2 h and activation temperature of 600 °C, in which the KOH to PMC ratios were varied at 2:1, 3:1, and 4:1. From Table 3 and Fig. 2a and b, an increase in the KOH to PMC ratio from 2:1 to 3:1 leads to substantially higher porosity (surface area, total pore volume, and micropore volume). A higher loading of KOH possibly resulted in a greater intercalation of potassium metal, generating an abundance of pores in the structure. However, a slight reduction in porosity (lower surface area and total pore volume) is observed for PMAC4-1–6 as the KOH to PMC ratio was increased to 4:1, suggesting that a further increase in the KOH loading did not enhance porosity but possibly caused minor pore collapse. Since the KOH to PMC ratio at 3:1 exhibits the highest porosity, this ratio is selected for the subsequent experiments.

As for the effect of activation time, the study was conducted under constant KOH to PMC ratio of 3:1 and activation temperature of 600 °C, with the variation of activation time being 1 h, 1,5 h, and 2 h. Based on Table 3 and Fig. 2c and d, an increase in activation time from 1 to 1.5 h resulted in a higher density of pores and micropores in PMAC3-1.5–6, as evidenced by its higher values of surface area, total pore volume, and micropore volume. The longer activation time allows for extensive pore creation due to intensified gasification and K_2_O/K_2_CO_3_ reduction reactions, as well as deeper potassium intercalation into the carbon layers. However, the prolonged activation of PMAC3-2–6 for a 2-h period causes an excessive activation situation, whereby pore wall collapse and pore enlargement from micropores to larger-sized micropores and mesopores possibly occurred, as showcased by its reduction in surface area, total pore volume, and micropore volume. Due to the highest porosity attained at an activation time of 1.5 h, this activation time is selected for the subsequent experiment.

The investigations on the effect of activation temperature were carried out at 500 °C, 600 °C, 700 °C under constant KOH to PMC ratio of 3:1 and activation time of 1.5 h. It is apparent from Table 3 and Fig. 2e and f that a steady increase in porosity is reflected by the increase in activation temperature from 500 to 700 °C. There was a 40% increase in total pore volume and a 34% increase in micropore volume of PMAC3-1.5–7 as compared to PMAC3-1.5–5. This signifies that higher activation temperature promotes new pore and micropore formations by enhancing their decomposition, gasification, and K_2_O/K_2_CO_3_ reduction reactions. The lower activation temperature is surmised to limit the etching of the carbon structure due to insufficient thermal energy (Yu et al. 2022).

#### SEM analysis

**Fig. 3:**
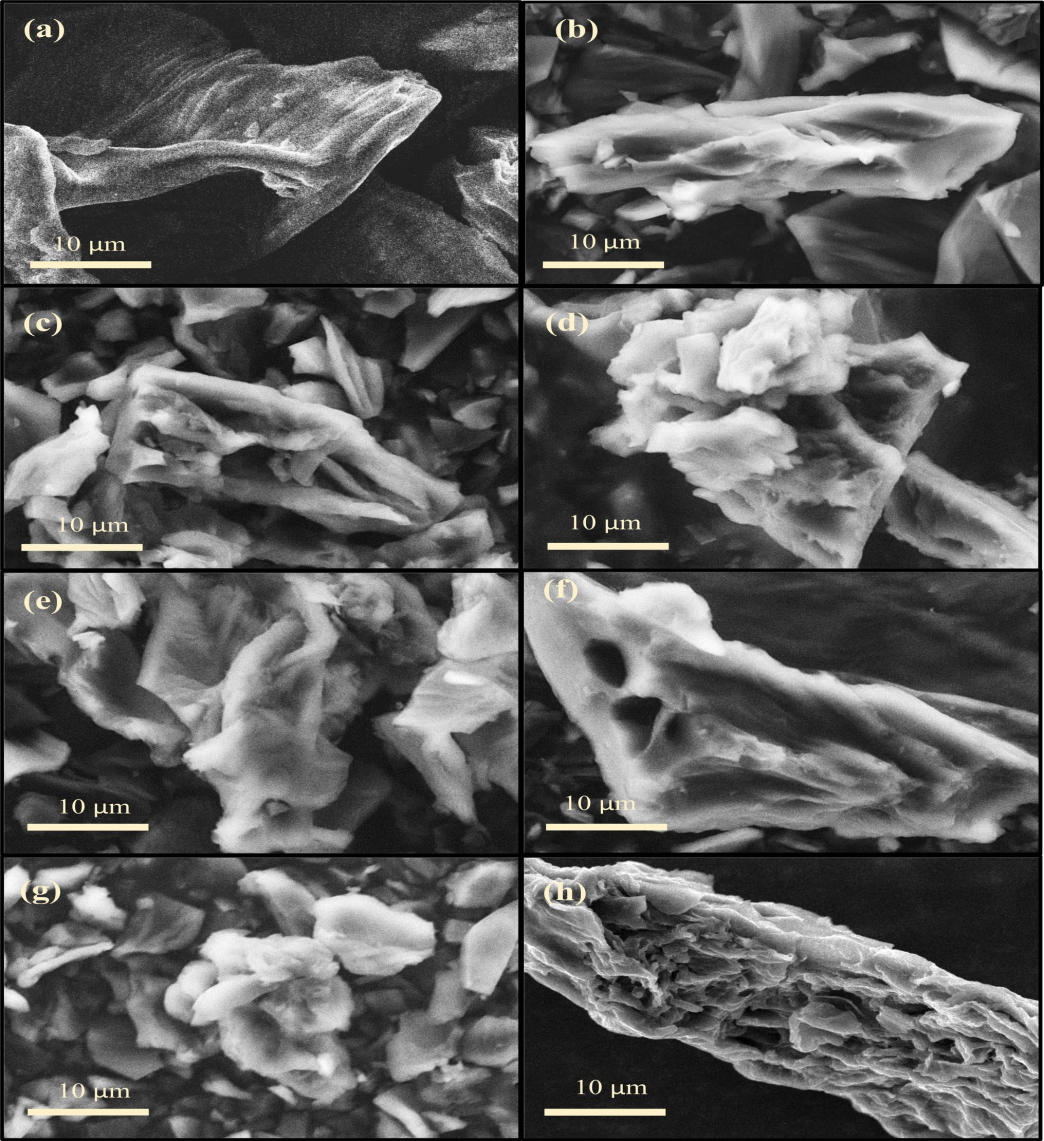
SEM image of (**a**) PM, (**b**) PMAC2-1–6, (**c**) PMAC3-1–6, (**d**) PMAC4-1–6, (**e**) PMAC3-1.5–6, (**f**) PMAC3-2–6, (**g**) PMAC3-1.5–5, and (**h**) PMAC3-1.5–7

The surface morphology image of PM and PMACs synthesised under various activation parameters is shown in Fig. 3. The SEM image of PM in Fig. 3a reveals a smooth surface with no visible irregularities. In contrast, the surface morphology images of PMACs in Fig. 3b–h generally show a rougher surface with ridges and pores. Additionally, the presence of irregular small fragments or particles on the surface of PMACs suggests structural breakdown from the carbonisation and KOH activation process. Although the BET analysis revealed differences in surface area and pore volume for each PMAC, these porosity variations are not distinguishable in the surface morphology images. This is probably because the pore structure of all PMACs is microporous-mesoporous, with micropores constituting the majority of the pore volume. These small pores are not observable in the SEM images. The only visible pores in some PMACs (PMAC-3-1−6, PMAC4-1–6, PMAC3-2–6, and PMAC3-1.5–7) are classified as larger macropores, which could potentially facilitate the diffusion of adsorbates into the inner network of micropores and mesopores. Notably, PMAC3-1.5–7 has the most observable pores amongst the PMACs, which likely corresponds to its having the highest porosity.

#### FTIR analysis

**Fig. 4:**
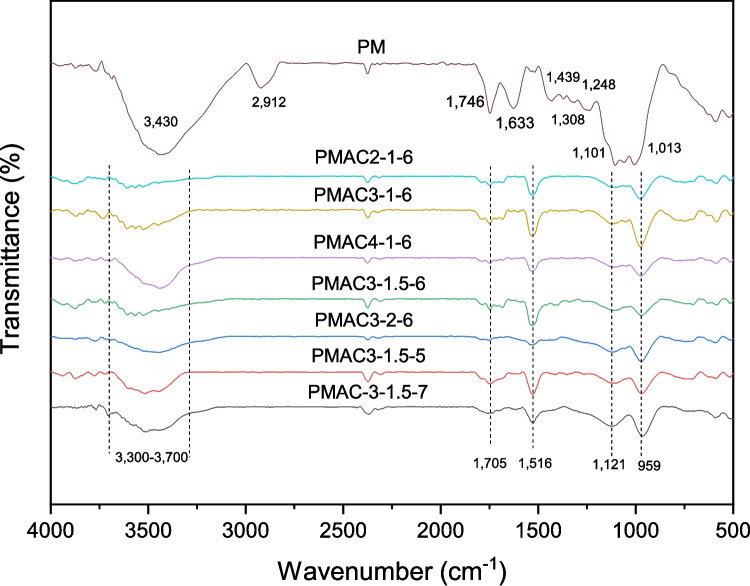
FTIR spectra of PM and PMACs

The surface functional groups of PM and PMACs were identified from the FTIR spectra as shown in Fig. 4. Firstly, the broad peak observed in PM’s spectrum at 3400 cm^−1^ indicates a strong presence of O-H stretching vibration from the cellulose and hemicellulose of PM and adsorbed water (Stavrinou et al. 2018). This broad peak was drastically reduced in intensity after the carbonisation and activation process, as depicted from the 3300 cm^−1^–3700 cm^−1^ band of PMACs. The reduction in intensity of this band is due to the reduction in the hydroxyl groups on the surface from a possible volatilisation and aromatisation process (Rashidi and Yusup 2017; Pallarés et al. 2018). Furthermore, a small peak in PM’s spectrum at 2912 cm^−1^ that denotes C-H stretching vibration in methyl group had disappeared in the spectrum of PMACs. This indicates the occurrence of C-H bond cleavage and dehydrogenation during the carbonisation and activation process. Besides the disappearance of the C-H stretching vibration peak, there were also the absence of several other peaks associated with C=O (1633 cm^−1^) and C-O (1308 cm^−1^ and 1248 cm^−1^) stretching vibration. These are due to the decomposition and volatilisation of the functional groups during the carbonisation and activation process. Instead, a newfound peak at 1516 cm^−1^ was identified on the PMACs. This peak represents the C=C stretching band in the aromatic ring and is probably resulted from the dehydration (Mo et al. 2016), dehydrogenation, and aromatisation reaction (Sher et al. 2020). In addition, the lower intensity of other peaks (1705 cm^−1^, 1121 cm^−1^, and 959 cm^−1^) on PMACs surmised loss of the related functional groups during the carbonisation and activation process. The small peak at 1705 cm^−1^ indicates C=O stretching vibration, whereas the two peaks at 1121 cm^−1^ and 959 cm^−1^ denote C-O or C-O-C stretching vibrations. Overall, it is inferred that the carbonisation and KOH activation process had largely decreased the oxygen and hydrogen-containing functional groups on PM, leading to a carbon rich structure of the PMACs.

#### XRD analysis

**Fig. 5:**
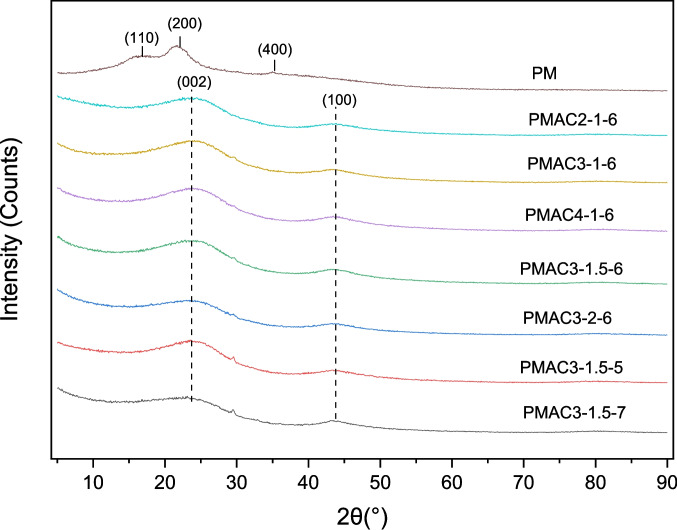
XRD diffractograms of PM and PMACs

The crystalline phases of PM and PMACs were evaluated through X-ray diffraction (XRD) diffractograms depicted in Fig. 5. From Fig. 5, it was found that there are three broad peaks at 16°, 22°, and 35° on the XRD pattern of PM, attributed to its crystalline cellulose I planes with Miller indices of (100), (200), and (400), respectively (He et al. 2022). As for the PMACs synthesised at various activation parameters, similar XRD patterns are exhibited for all of the PMACs—two broad peaks at around 24° and 43°. These peaks are identified as (002) and (100) graphitic carbon planes, respectively (Singh et al. 2021). The broadness of the peaks demonstrates the low degree of graphitic character in the PMACs, indicating that the structure of the PMACs probably consists of randomly oriented graphitic carbon planes. These disordered graphitic carbon planes in the structure of the PMACs potentially contribute to formation of edges, defects, and pores within the PMACs. As a result, abundant active sites are expected, consequently resulting in a possible high adsorption potential for the PMACs. Another noteworthy observation is that the peaks broadened as the activation temperature increased from 500 °C (PMAC3-1.5–5) to 700 °C (PMAC3-1.5–7), indicating that a higher activation temperature leads to an increase in structural disorder. Similarly, the more pronounced flattening of the peaks observed with the increase in activation time from 1.5 h (PMAC3-1.5–6) to 2 h (PMAC3-2–6) also suggests an increase in structural disorder due to the longer activation duration. A comparison of the XRD patterns of all PMACs highlighted the appearance of a small sharp peak at around 29° on PMAC3-1–6, PMAC3-1.5–5, and PMAC3-1.5–7. The presence of this peak on several of the PMACs is likely due to the potassium chloride (KCl) salt compound left from the acid-base reaction of KOH and HCl during the washing procedure (Quan et al. 2020a). It is worth noting that the existence of this salt compound in the activated carbon could cause pore blockage, which may affect the measured surface area and adsorption capacity of the samples. Thus, multiple sequential washing cycles procedure is necessary in future work to ensure complete removal of the residual salts as stated in Section “Chemical activation of pomelo peel-based biochar”.

#### TGA-DTG analysis

**Fig. 6:**
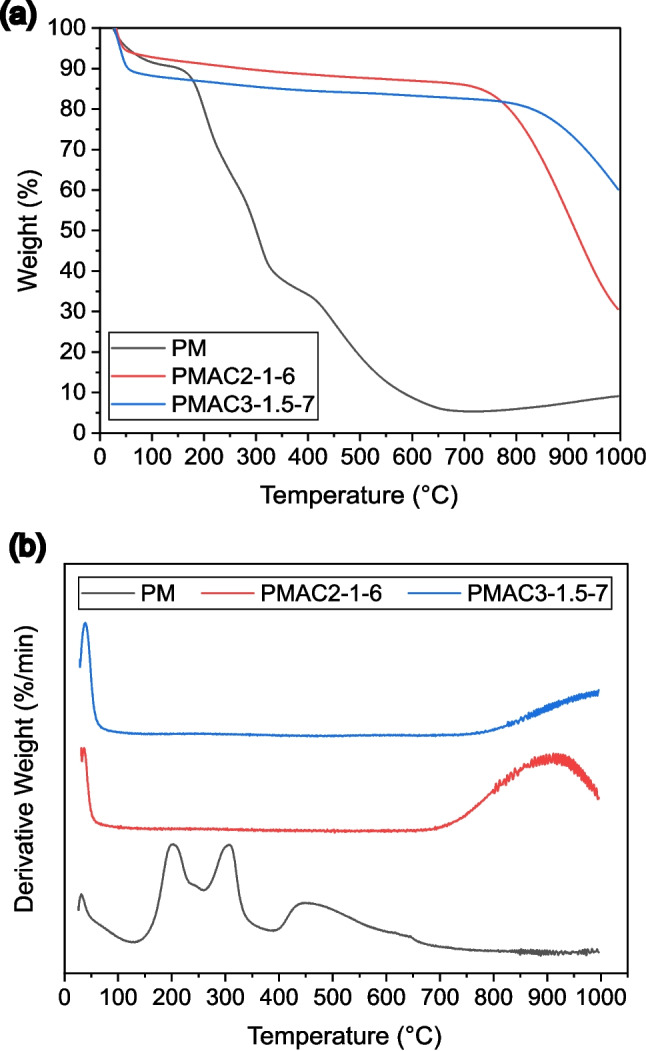
(**a**) TGA and (**b**) DTG plot of PM, PMAC2-1–6, and PMAC3-1.5–7

The thermogravimetric analysis (TGA) and derivative thermogravimetric (DTG) plots of PM, PMAC2-1–6, and PMAC3-1.5–7 in Fig. 6 showcase the profiles of weight loss and the rate of weight loss of the samples as functions of temperature, respectively. PMAC2-1–6 and PMAC3-1.5–7 were selected for this analysis to compare the thermal stability of the PMAC with the lowest surface area (PMAC2-1–6) and the highest surface area (PMAC3-1.5–7). Based on these plots, PM exhibited a significantly larger weight loss compared to PMAC2-1–6 and PMAC3-1.5–7, whereby the total weight loss of PM accumulated to 93% of the sample’s initial weight. The DTG curve of PM consists of 4 degradation peaks at temperatures of 32 °C, 202 °C, 308 °C, and 450 °C. The first degradation peak at 32 °C is attributed to the removal of water molecules from the PM, which occurs at temperatures below 120 °C. The subsequent major degradation (~ 85%) of PM between 120 and 670 °C demonstrates the volatile matter decomposition in cellulose, hemicellulose, and lignin components. The degradation peaks at 202 °C and 308 °C are possibly due to the decomposition of cellulose and hemicellulose (Huang et al. 2019; Sai Prasanna and Mitra 2020), whereas the peak at a higher temperature of 450 °C is most likely attributed to lignin decomposition (Giraldo and Moreno-Piraján 2018; Huang et al. 2019). The char formed from continuous heating until 1000 °C is calculated to be around 9% of PM’s original weight.

The comparison of TGA and DTG plots between PMAC2-1–6 and PMAC3-1.5–7 shows that the latter possesses higher thermal stability than the former. This is evidenced by the lower total weight loss of PMAC3-1.5–7 (~ 40%) as compared to PMAC2-1–6 (~ 60%). Both PMAC samples exhibit a similar trajectory profile for TGA and DTG plots, with initial water elimination occurring below 100 °C and a later major weight loss within the higher temperature region. However, there is an obvious difference in the onset temperature of the major weight loss, with a higher onset temperature observed for PMAC3-1.5–7 (750 °C) as compared to PMAC2-1–6 (650 °C). This further demonstrates the higher thermal resistance of PMAC3-1.5–7 compared to PMAC3-2–6. The weight loss within a higher temperature region in PMACs is a result of the decomposition of the remaining lignin and other organic compounds that have not fully decomposed during the carbonisation and KOH activation process (Moreno-Barbosa et al. 2013; Giraldo and Moreno-Piraján 2018). It is also worth noting that a higher onset temperature for PMAC3-1.5–7 in comparison to PMAC2-1–6 stemmed from the higher activation temperature of PMAC3-1.5–7 during the KOH activation process.

### Effect of activation parameters on CO_2_ adsorption performance

**Fig. 7:**
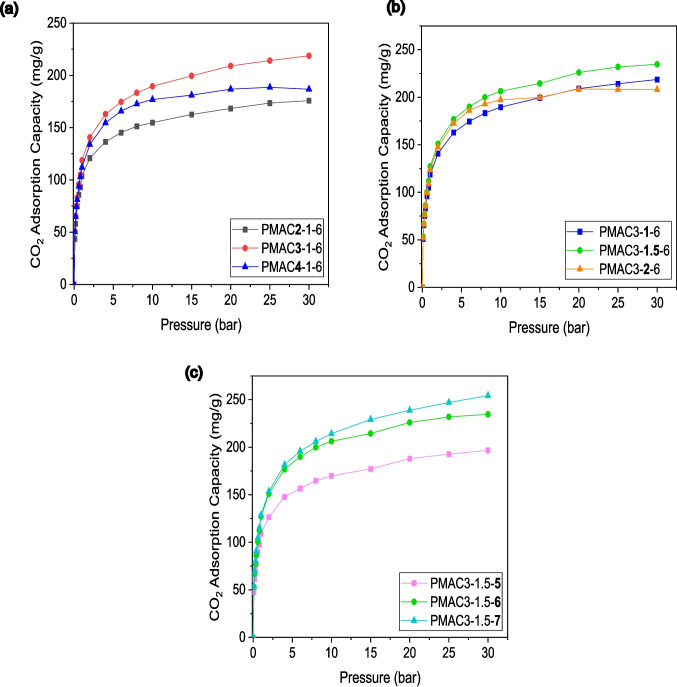
CO_2_ adsorption isotherm at 25 °C for PMACs synthesised under various (**a**) KOH to PMC ratio, (**b**) activation time, and (**c**) activation temperature

The pure CO_2_ adsorption performance of PMAC adsorbents synthesised under various activation parameters (KOH to PMC mass ratio, activation time, and activation temperature) is depicted in Fig. 7. The CO_2_ adsorption isotherms of the PMAC adsorbents in Fig. 7 exhibit similar shapes, featuring a rapid increase in adsorption at low pressure followed by a gradual adsorption increment at high pressure. This adsorption isotherm shape resembles a typical type I isotherm according to the IUPAC classification.

The investigation into the effect of activation agent ratio (KOH to PMC mass ratio) on CO_2_ adsorption performance of the PMAC adsorbent in Fig. 7a reveals that an increase in the KOH to PMC mass ratio from 2:1 to 3:1 significantly enhances CO_2_ uptake for PMAC3-1–6 at every pressure reading. As can be seen, PMAC3-1–6 attained a maximum CO_2_ uptake of 218.7 mg/g at 30 bar, compared to 175.7 mg/g for PMAC2-1–6. This substantial increment is mainly attributed to the higher BET surface area, total pore volume, and micropore volume of PMAC3-1–6 (386.4 m^2^/g, 0.20 cm^3^/g, and 0.13 cm^3^/g) compared to PMAC2-1–6 (170.5 m^2^/g, 0.09 cm^3^/g, and 0.07 cm^3^/g). However, a further increase in the KOH to PMC mass ratio to 4:1 is observed to cause a reduction in CO_2_ uptake of PMAC4-1–6, possibly due to its lower BET surface area (383.7 m^2^/g) and total pore volume (0.19 cm^3^/g). This suggests that the utilisation of superfluous KOH activation agent can result in a decline in porosity, thereby reducing CO_2_ adsorption capacity of the adsorbent. The abundance of pores plays a crucial role in providing energetically favoured adsorption sites for CO_2_ gas molecules.

Figure 7b compares the CO_2_ adsorption performance of PMAC adsorbents synthesised with different activation times (1 h, 1.5 h, and 2 h). Amongst these adsorbents, PMAC3-1.5–6.5, which was activated for 1.5 h, demonstrated the highest CO_2_ uptake. The maximum adsorption capacity of PMAC3-1.5–6.5 at 30 bar is 234.6 mg/g, notably higher than 218.7 mg/g and 208.1 mg/g for PMAC3-1–6 and PMAC3-2–6, respectively. The evident reason for this is its higher BET surface area (392.4 m^2^/g), total pore volume (0.21 cm^3^/g), and micropore volume (0.14 cm^3^/g) compared to PMAC3-1–6 and PMAC3-2–6. An increase in activation time to 2 h led to a decrease in CO_2_ adsorption performance of PMAC3-2–6. This reduction was attributed to possible pore collapse, which resulted in decreased BET surface area (356.9 m^2^/g), total pore volume (0.18 cm^3^/g), and micropore volume (0.12 cm^3^/g). Similar to the effect of the activation agent mass ratio, an overextended activation time can also have a negative effect on the porosity of the adsorbent and reduce its CO_2_ adsorption performance.

From Fig. 7c, the increase in the activation temperature from 500 to 700 °C of the PMAC adsorbents has produced a steady rise in CO_2_ adsorption performance. As can be seen, at a pressure of 30 bar, there is a significant increase in adsorption capacity from 196.8 mg/g for PMAC3-1.5–5 to 254.3 mg/g for PMAC3-1.5–7. This increasing trend of CO_2_ adsorption capacity with activation temperature results from an increase in overall porosity (BET surface area and total pore volume) and microporosity (micropore volume). Amongst the PMAC adsorbents studied, PMAC3-1.5–7, synthesised with a KOH:PMC mass ratio of 3:1, activation time of 1.5 h, and activation temperature of 700 °C, exhibited the highest CO_2_ uptake. The summarised CO_2_ adsorption capacity values of the PMAC adsorbents are found in Table 3.

Based on the analysis of CO_2_ adsorption performance of the PMAC adsorbents, it can be inferred that the variation of all activation parameters has substantial impacts on the adsorption of CO_2_ gas molecules. The main properties of PMAC adsorbents that directly correlate with CO_2_ adsorption uptake are their pore properties—BET surface area, total pore volume, and micropore volume. Numerous previous studies have also supported this, highlighting microporosity as the main contributor to the high CO_2_ uptake of carbon-based adsorbents, especially at low adsorption pressures (Fiuza-Jr et al. 2016; Yaumi et al. 2017; Shen et al. 2018; Serafin et al. 2022). Besides micropores, mesopores, which also contribute to the overall BET surface area and total pore volume of the PMAC adsorbents, play an important role in speeding up CO_2_ diffusion into the pores and adsorbing CO_2_ at high adsorption pressures.

The CO₂ uptake of PMAC-3-1.5–7 is compared with activated carbons from previous studies in Table 4. The comparisons were conducted under standard conditions of 25 °C and 1 bar, as most CO₂ adsorption performances are evaluated under these parameters. PMAC-3-1.5–7 is a competitive adsorbent, demonstrating superior CO₂ adsorption performance compared to activated carbons derived from waste precursors such as rubber seed shell, PET waste, bamboo, palm kernel shells, tea seed shell (doped with melamine), as well as commercial activated carbon Norit SX2. Furthermore, the CO₂ adsorption performance of PMAC-3-1.5–7 is comparable to that of activated carbon derived from another citrus fruit peel, orange peel. However, PMAC-3-1.5–7 exhibits lower CO₂ adsorption performance than activated carbons derived from waste distiller’s grain, corn starch (doped with thiourea), and coconut shell (doped with melamine), which can be attributed to their higher specific surface areas and heteroatom doping (sulphur and nitrogen).

**Table 4:** Comparison of CO_2_ adsorption performances of various activated carbons

Activated carbon source	BET specific surface area (m^2^/g)	CO_2_ adsorption capacity at 25 °C and 1 bar (mg/g, mmol/g)	Reference
PMAC3-1.5–7(pomelo peel)	455.5	128.7 (2.90)	This study
Rubber seed shell	1129.7	85.43 (1.94)	Borhan et al. (2019)
PET waste	1690	101.6 (2.31)	Kaur et al. (2019)
Waste distiller’s grain	1059	187.4 (4.26)	Luo et al. (2022)
Corn starch (thiourea-doped)	1795	188.2 (4.28)	Nazir et al. (2021)
Bamboo	787.0	110.9 (2.52)	Dilokekunakul et al. (2020)
Orange peel	457.2	134.0 (3.05)	Deepak et al. (2023)
Coconut shell (urea-doped)	1082	163.2 (3.71)	Yue et al. (2018)
Date seeds	798.4	129.2 (2.93)	Ogungbenro et al. (2018)
Tea seed shell (melamine-doped)	1187.9	121.0 (2.75)	Quan et al. (2020a)
Palm kernel shell	303.9	93.7 (2.13)	Rashidi and Yusup (2018)
Commercial activated carbon Norit SX2	654	82.7 (1.88)	Rashidi and Yusup (2018)

### RSM analysis for adsorption process parameters

The effects of CO_2_ adsorption process parameters (temperature, pressure, and CO_2_ composition in CO_2_-N_2_ gas mixture) and their interactions on the adsorption capacity of PMAC3-1.5–7 are investigated through response surface methodology (RSM) modelling. The experimental design, utilising Box-Behnken design (BBD), along with the values of the predicted and actual response variable (adsorption capacity), are shown in Table 5. The quadratic model fitted to the experimental data is represented by Eq. 9, where *A*, *B*, and *C* represent temperature, pressure, and CO_2_ composition, respectively.

**Table 5:** BBD design matrix and its responses

Experiment No	Independent variables			Adsorption capacity (mg/g)	
	A: Temperature (°C)	B: Pressure (bar)	C: CO_2_ composition (%)	Experimental	Predicted
1	45	20	30	144.015	145.6667
2	45	30	10	112.482	113.7585
3	65	20	50	151.833	152.3554
4	45	20	30	147.390	145.6667
5	25	30	30	179.828	179.074
6	65	30	30	141.877	139.7492
7	25	20	50	193.957	193.1066
8	45	20	30	145.536	145.6667
9	65	20	10	90.195	91.04517
10	45	10	50	145.133	143.8567
11	45	30	50	175.129	176.7351
12	45	20	30	146.764	145.6667
13	45	20	30	145.031	145.6667
14	45	10	10	84.921	83.31527
15	65	10	30	106.357	107.1108
16	25	10	30	146.263	148.3908
17	25	20	10	131.421	130.8988

9 $$\text{Adsorption Capacity }\left(\mathrm{mg}/\mathrm{g}\right)=93.22419-2.20394A+4.28586B+2.85684C+0.002444AB+0.000561AC+0.003044BC+0.012937{A}^{2}-0.072603{B}^{2}-0.022475{C}^{2}$$

The result of the analysis of variance (ANOVA) for the quadratic model is presented in Table 6. Based on the model’s *p*-value and *F*-value, it is deemed statistically significant, as its *F*-value (373.96) is large and its *p*-value (< 0.0001) is less than 0.05. Additionally, the *p*-value of the lack of fit (LOF) for the model exceeds 0.005, indicating a high probability that the model does not deviate significantly from the experimental data, further affirming its adequacy in fitting the data. The fitting statistics of the model, such as the coefficient of determination (*R*^2^), adjusted *R*^2^, and predicted *R*^2^, are shown in Table 6. The *R*^2^ value of the quadratic model, calculated at 0.9979, demonstrates a strong fit between the model and the experimental data (i.e. the *R*^2^ value is close to 1). Furthermore, the small difference between the adjusted *R*^2^ (0.9953) and predicted *R*^2^ (0.9749), being less than 0.2, suggests good model generalisability due to their close agreement.

**Table 6:** ANOVA and fitting statistics of the quadratic model

Source	Coded coefficient	Sum of squares	df	*F*-value	*p*-value	Remarks
Model		13,554.15	9	373.96	< 0.0001	Significant
A-Temperature	−20.15	3248.46	1	806.63	< 0.0001	Significant
B-Pressure	15.83	2004.77	1	497.81	< 0.0001	Significant
C-CO_2_ composition	30.88	7628.16	1	1894.15	< 0.0001	Significant
*AB*	0.4887	0.9555	1	0.2373	0.6411	Not significant
*AC*	−0.2245	0.2016	1	0.0501	0.8293	Not significant
*BC*	0.6087	1.48	1	0.3681	0.5632	Not significant
*A*^2^	5.17	112.76	1	28.00	0.0011	Significant
*B*^2^	−7.26	221.95	1	55.11	0.0001	Significant
*C*^2^	−8.99	340.30	1	84.50	< 0.0001	Significant
Residual		28.19	7			
Lack of fit (LOF)		20.60	3	3.62	0.1231	Not significant
Pure error		7.59	4			
*R*^2^	0.9979					
Adj *R*^2^	0.9953					
Predicted *R*^2^	0.9749					

**Fig. 8:**
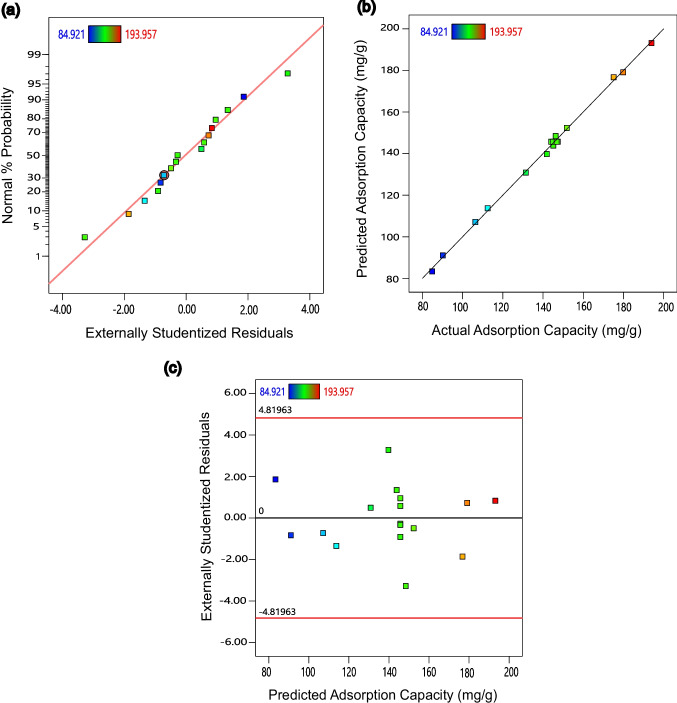
Plot of (**a**) normal probability, (**b**) predicted vs. actual adsorption capacity, and (**c**) residual vs. predicted adsorption capacity

The diagnostic plots (normal probability plot, predicted vs. adsorption capacity plot, and residuals vs. prediction plot) shown in Fig. 8 are used to further assess the fitness and reliability of the model. In Fig. 8(a), deviations from normality can be diagnosed through the distribution of residuals (i.e. errors). Since most of the residuals closely align with the straight line, indicating a normal distribution, the reliability of the model is affirmed. Moreover, the predicted vs. actual adsorption capacity plot in Fig. 8(b) demonstrates that the model accurately predicts adsorption capacity, with points falling near or on the straight line. The residuals vs. predicted adsorption plot in Fig. 8(c) assesses the adequacy of the model by observing the scatter of residuals. This plot shows random scatter of residuals without outliers, suggesting constant variances and good fit between the model and experimental data. All of these results indicate that the quadratic model is statistically accurate, adequate, and reliable.

From the ANOVA results in Table 6, significant terms in the quadratic model are identified based on their *p*-values; terms with a *p*-value less than 0.05 are considered statistically significant. Hence, terms *A*, *B*, *C*, *A*^2^, *B*^2^, and *C*^2^ are deemed statistically significant, while the interaction terms of the two parameters (*AB*, *AC*, and *BC*) are considered statistically insignificant. Since the interaction terms are regarded as insignificant, they are excluded from the final Eq. 10 for the quadratic model.

10 $$\text{Adsorption Capacity }\left(\mathrm{mg}/\mathrm{g}\right)=93.22419-2.20394A+4.28586B+2.85684C+0.012937{A}^{2}-0.072603{B}^{2}-0.022475{C}^{2}$$

**Fig. 9:**
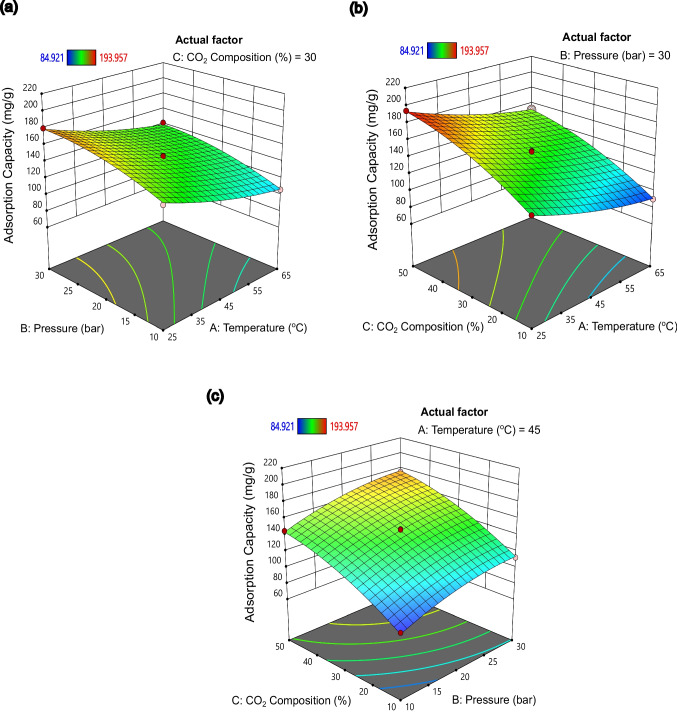
3D response surface plot of adsorption capacity as a function of (**a**) temperature and pressure, (**b**) temperature and CO_2_ composition, and (**c**) pressure and CO_2_ composition

Based on the coded coefficient magnitudes of the terms in Table 6, the order of relative impacts on adsorption capacity is as follows: *C* > *A* > *B* > *C*^2^ > *B*^2^ > *A*^2^. The individual parameter with the highest relative impact is CO_2_ composition, followed by temperature and pressure. The effects of these parameters on adsorption capacity are further illustrated in the 3D surface response plots in Fig. 9. The first 3D plot in Fig. 9a shows the relationship between adsorption capacity and the two parameters (temperature and pressure), indicating an increasing trend in adsorption capacity with decreasing temperature and increasing pressure. In Fig. 9b, there appears to be favourable adsorption with lower temperature and higher CO_2_ composition. Furthermore, in the 3D plot of Fig. 9c, positive correlations between both CO_2_ composition and pressure with adsorption capacity are exhibited. Overall, these 3D plots conclusively demonstrate that the adsorption capacity of PMAC3-1.5–7 is enhanced with an increase in pressure and CO_2_ composition, while it decreases with increasing temperature.

The observed inverse relationship between temperature and adsorption capacity is one of the characteristics consistent with the adsorption being predominantly governed by a physical adsorption process (Mudoi et al. 2022). Adsorption at higher temperatures may result in partial desorption of these gases due to disruption of the weak intermolecular interactions between the adsorbent and gas molecules caused by increased thermal energy. These relatively weak interactions (compared to chemical bond formation) may include dispersion forces as well as localised electrostatic interactions (dipole–quadrupole and hydrogen bonding) between gas molecules and the oxygen-containing functional groups on the adsorbent surface (Chiang et al. 2019; Cheng et al. 2025). At lower temperatures, the lower kinetic of gas molecules can induce more adherence and trapping of gas adsorbates onto the adsorbent due to the slower gas molecule movement. This inverse relationship trend is also the inherent general characteristic of the adsorption being an exothermic process (Singh et al. 2017; Karimi et al. 2018).

As for the increasing trend in adsorption capacity of PMAC3-1.5–7 with pressure, this is primarily due to the greater availability of gas adsorbates to interact with the surface of the adsorbent as well as the higher driving force for adsorption created by the difference between the concentration of gas in the bulk phase and at the surface. This increase in adsorption capacity with pressure will eventually plateau at a much higher pressure than the range of values studied in the RSM modelling, due to the saturation of the adsorbent.

The positive correlation between the CO_2_ composition in the gas mixtures (CO_2_ and N_2_) and the adsorption capacity of PMAC3-1.5–7 is attributed to the stronger interaction between CO_2_ molecules and the surface of the adsorbent compared to N_2_ molecules. The higher polarisability and quadrupole moments of CO_2_, as compared to N_2_ molecules, result in CO_2_ occupying adsorption sites that N_2_ cannot strongly adsorb to (Boonchuay and Worathanakul 2022). Therefore, a higher CO_2_ concentration can lead to the utilisation of more adsorption sites, consequently increasing the overall adsorption capacity of the adsorbent.

The numerical optimisation in the Design Expert Software was carried out based on the main goal of maximising the adsorption capacity of the adsorbent. The optimised parameter’s value that was provided by the software is 25 °C for temperature, 30 bar for pressure, and 50% for CO_2_ composition, resulting in a predicted adsorption capacity value of 201.796 mg/g. The verification of the predicted value was confirmed through experimental work, where triplicate adsorption runs were conducted under the optimised conditions. The adsorption capacity values from these runs are presented in Table 7, along with the average value. The experimental runs yielded an average actual adsorption capacity of 203.031 mg/g. This value is considered very close to the predicted optimised value, with a percentage difference of approximately 0.608%, which further confirms the prediction accuracy of the quadratic model. The optimised parameter values obtained in this study for pomelo peel-based activated carbon align with those reported for strontium hydroxide-modified nanoclay montmorillonite, whereby the optimised adsorption temperature and pressure were also at the lowest and highest values, respectively, within the studied parametric range (Khajeh and Ghaemi 2021). A study by Saeidi et al. (2020) on potassium hydroxide adsorbent similarly observed an optimal adsorption temperature at its lowest value. However, the optimal adsorption pressure for this adsorbent was identified at 6 bar within a studied range of 2 to 10 bar (Saeidi et al. 2020). The variations in the optimal adsorption process parameters across different studies are attributed to the specific adsorbent material properties and their unique adsorption mechanisms.

**Table 7:** Actual and predicted value of the adsorption capacity at optimised conditions

Replicate	Adsorption capacity (mg/g)	
	Actual	Predicted
1	203.821	201.796
2	201.019	
3	204.253	
Average	203.031	

## Conclusion

This study successfully demonstrates that pomelo peel-based activated carbon (PMAC) is a potentially effective adsorbent for CO₂ capture. The KOH activation process parameters were shown to significantly influence the textural properties and CO₂ adsorption performance of PMAC, with PMAC3-1.5–7 (3:1 KOH:PMC, 1.5 h, and 700 °C) exhibiting the highest porosity and CO₂ adsorption capacity across a wide pressure range. The optimisation of adsorption process parameters through RSM modelling identified the optimal adsorption conditions at 25 °C, 30 bar, and 50% CO₂, with adsorption capacity being negatively influenced by increasing temperature and positively influenced by increasing pressure and CO₂ composition. Overall, these findings underscore the potential of biowaste valorisation as a low-cost and sustainable approach for producing CO₂ adsorbents, while also providing valuable guidelines on the tailoring of activation and adsorption process parameters for practical industrial applications. In future works, it will be important to carry out adsorption-desorption cyclic tests to better understand the regenerability and long-term stability of PMAC. Likewise, fixed-bed dynamic breakthrough experiments can be conducted to capture its column adsorption behaviour under realistic operating conditions. Evaluating performance in humidified flue gas environments will also provide crucial insights into the practical viability of PMAC for industrial CO₂ capture.

## Data Availability

The datasets generated and analysed during the current study are not publicly available due to institutional policy restrictions and ongoing related research. However, summary data supporting the findings of this study are available from the corresponding author upon reasonable request.
